# Serum Proteomic Profiling Identifies ACSL4 and S100A2 as Novel Biomarkers in Feline Calicivirus Infection

**DOI:** 10.3390/ijms27021047

**Published:** 2026-01-21

**Authors:** Chunmei Xu, Hao Liu, Haotian Gu, Di Wu, Xinming Tang, Lin Liang, Shaohua Hou, Jiabo Ding, Ruiying Liang

**Affiliations:** 1 Key Laboratory of Animal Biosafety Risk Prevention and Control (North) & Key Laboratory of Veterinary Biological Products and Chemical Drugs of MARA, Institute of Animal Science, Chinese Academy of Agricultural Sciences, Beijing 100193, China; xcm13592563704@163.com (C.X.); 18593435727@163.com (H.L.); 15052528858@163.com (H.G.); tangxinming@caas.cn (X.T.); lianglin@caas.cn (L.L.); houshaohua@caas.cn (S.H.); dingjiabo@caas.cn (J.D.); 2 Beijing Center for Animal Disease Prevention and Control, Beijing 102629, China; 13651007346@163.com

**Keywords:** feline calicivirus, serum proteomics, biomarker, ACSL4, S100A2

## Abstract

Feline calicivirus (FCV) is a highly variable RNA virus that infects domestic cats and circulates endemically within feline populations, causing a wide spectrum of clinical manifestations, from asymptomatic infections to severe disease. Genomic analysis of 69 FCV strains revealed a high prevalence of the virus across multiple provinces in China. In vitro infection of CRFK cells with laboratory isolates FCV-BJ616 and FCV-BJDX40 resulted in significant cytotoxic effects. Serum proteomic analysis identified 221 upregulated and 123 downregulated proteins following infection with FCV-BJ616, and 233 upregulated and 165 downregulated proteins following infection with FCV-BJDX40. Among these, 215 proteins exhibited shared differential expression. Functional analyses revealed enriched pathways, including TNF signaling and ferroptosis. Notably, upregulation of Acyl-CoA Synthetase Long-Chain Family Member 4 (ACSL4) was correlated with lung injury, while downregulation of S100 Calcium Binding Protein A2 (S100A2) was associated with poor prognosis in FCV-associated oral disease. The differential expression of ACSL4 and S100A2 was further validated through Western blot analysis. These results suggest that ACSL4 and S100A2 are promising candidate biomarkers for monitoring FCV infection and disease progression, laying a foundation for future diagnostic and prognostic applications.

## 1. Introduction

Feline calicivirus (FCV) is a highly mutable RNA virus that poses a significant threat to domestic cats due to its high transmissibility and infectivity [1,2,3]. Although FCV infections typically manifest as oral and upper respiratory tract diseases (URTDs), the virus exhibits significant genetic and antigenic variability, which results in varied tissue tropism among different strains [4,5,6]. The lungs are a critical target organ, and FCV infection can cause pulmonary lesions that range from mild to life-threatening, including severe pneumonia. In virulent systemic disease (VSD), the primary fatal manifestation is interstitial bronchopneumonia [7], frequently accompanied by subcutaneous edema and necrosis in multiple organs, such as the pancreas, liver, and spleen [8,9,10]. Notably, young cats infected with FCV risk developing fatal pneumonia, even without progressing to full-blown VSD. Therefore, assessing pulmonary damage is crucial for evaluating the severity and prognosis of FCV infections [11,12].

Data-independent acquisition (DIA) quantitative proteomics, in combination with liquid chromatography-tandem mass spectrometry (LC-MS/MS), has been extensively employed to analyze host responses to viral infections across humans, animals, and plants [13,14,15]. Furthermore, DIA-based quantitative proteomics facilitates the identification of key protein biomarkers that aid in early disease detection, diagnosis, monitoring, and therapeutic management [15]. For instance, by integrating multi-omics analysis, this study elucidated host protein expression patterns in patients with acute respiratory distress syndrome (ARDS) and identified a specific set of serum proteins as prognostic indicators [16]. Additionally, the analysis of urine proteins in hereditary angioedema and the identification of serum biomarkers in COVID-19 patients demonstrate the potential of proteomics in uncovering disease mechanisms and enhancing clinical diagnosis [17,18]. A comparative analysis of different genotypes of the hepatitis B virus using DIA quantitative proteomics has further broadened the research scope of biomarkers [19]. Collectively, these studies underscore the significant role of proteomics in early warning, diagnosis, and personalized treatment strategies. Consequently, serum proteomics provides a comprehensive profile of host responses during viral infections and facilitates in-depth investigations into dysregulated signaling pathways, thereby elucidating the underlying molecular pathogenesis. However, to our knowledge, quantitative serum proteomic analysis has not yet been applied to investigate host responses to FCV infection.

In China, the prevalence of FCV has shown a concerning gradual increase over the past five years [20,21,22]. However, the systemic host response and underlying pathogenic mechanisms, particularly those detectable in serum, remain poorly understood. To address this gap, we hypothesize that FCV infection induces specific changes in the host serum proteome, potentially revealing novel biomarkers associated with mechanisms such as ferroptosis. Based on our preliminary evidence, we propose that biomarkers ACSL4 and S100A2 represent potential candidates to explore for enhancing the diagnosis and prognosis of this important viral disease.

## 2. Results

### 2.1. FCV Infection Causes Severe Lung Damage in Cats

**Figure 1:**
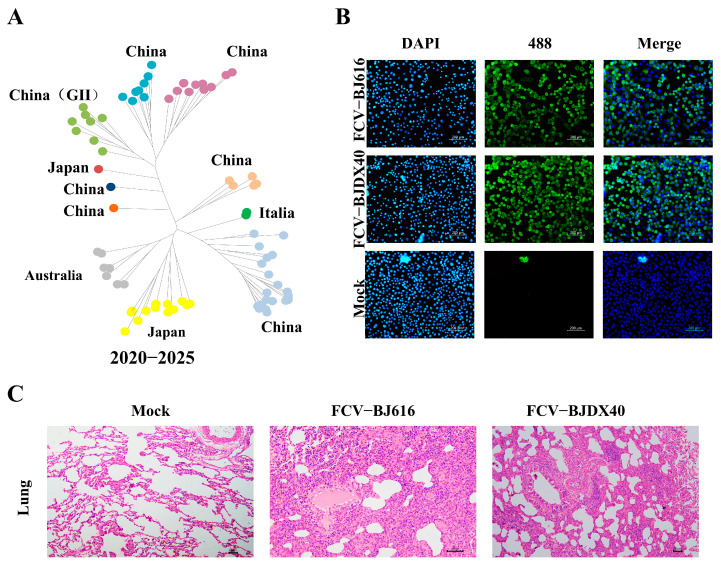
FCV causes severe lung damage. (**A**) The geographical distribution map shows that the samples are mainly from China, Japan, Italy, and Australia. (**B**) Immunofluorescence detection of FCV VP1 (green fluorescent marker) expression in CRFK cells infected with FCV-BJ616 and FCV-BJDX40. Scale bar = 200 μm. (**C**) Histopathological characteristics of cat lungs infected with FCV-BJ616 and FCV-BJDX40. Scale bar = 50 μm.

Geospatial analysis of 69 FCV strains (NCBI database, 2020–2025) identified China as a region of elevated prevalence (Figure 1A). To further investigate the pathogenic potential of FCV strains from this hotspot, we selected two recent clinical isolates, BJ616 and BJDX40, for in vitro and in vivo characterization. Infection of CRFK cells with these strains was confirmed by immunofluorescence staining against the VP1 capsid protein (Figure 1B). Consistent with their in vitro infectivity, histopathological examination of infected cats revealed that both strains induced severe pulmonary lesions, characterized by marked thickening of alveolar walls and extensive inflammatory cell infiltration (Figure 1C).

### 2.2. DIA Sample Quality Control

**Figure 2:**
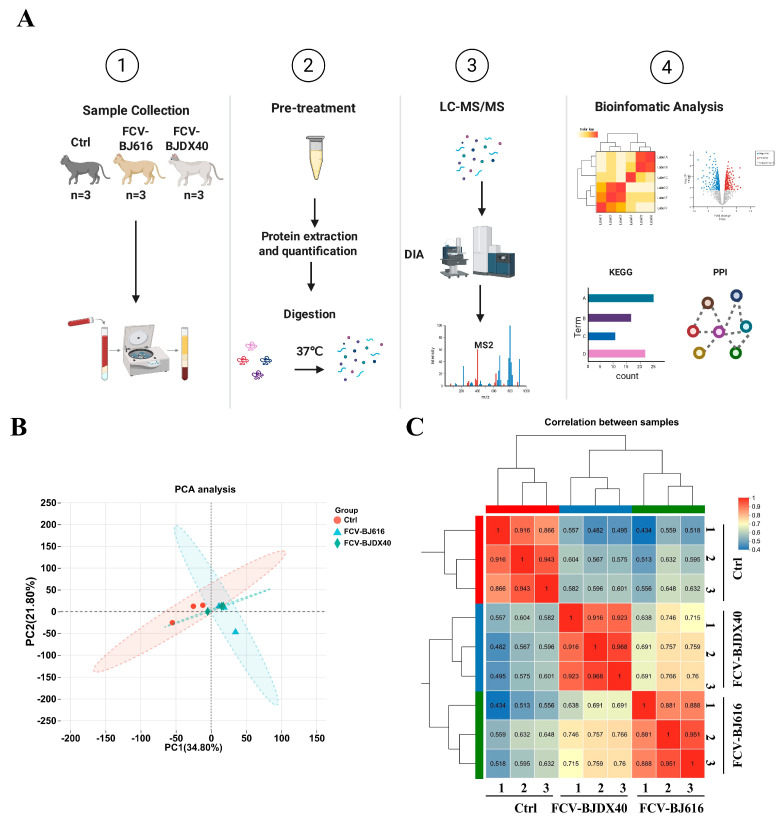
Seroproteomic analysis of cats infected with FCV. (**A**) Flowchart of DIA proteomic analysis in serum from cats infected with FCV-BJ616 and FCV-BJDX40. (**B**) PCA analysis of serum samples from cats infected with FCV-BJ616 and FCV-BJDX40 (*n* = 3 per group). (**C**) Heatmap of differential protein correlations in serum from cats infected with FCV-BJ616 and FCV-BJDX40. Red indicates positive correlation, blue indicates negative correlation, with darker colors representing stronger correlations.

This study aimed to identify and analyze proteins associated with FCV infection by extracting proteins from cat sera before and after infection with FCV-BJ616 and FCV-BJDX40, followed by DIA analysis. The main experimental workflow is illustrated in Figure 2A. Principal component analysis (PCA) was performed on the quantitative proteomic data of all samples. PCA demonstrated intragroup sample clustering with partial intergroup overlap, indicating methodological consistency (Figure 2B). Inter-sample correlation heatmaps further validated data reproducibility (Figure 2C). These QC metrics confirm the robustness of serum proteomics data for differential protein analysis. These results indicate that the data obtained from the serum samples are of high quality and reproducibility, providing a reliable foundation for subsequent differential gene analysis.

### 2.3. Differential Protein Expression and Interaction Analysis of Cat Sera Infected with FCV-BJ616 and FCV-BJDX40

**Figure 3:**
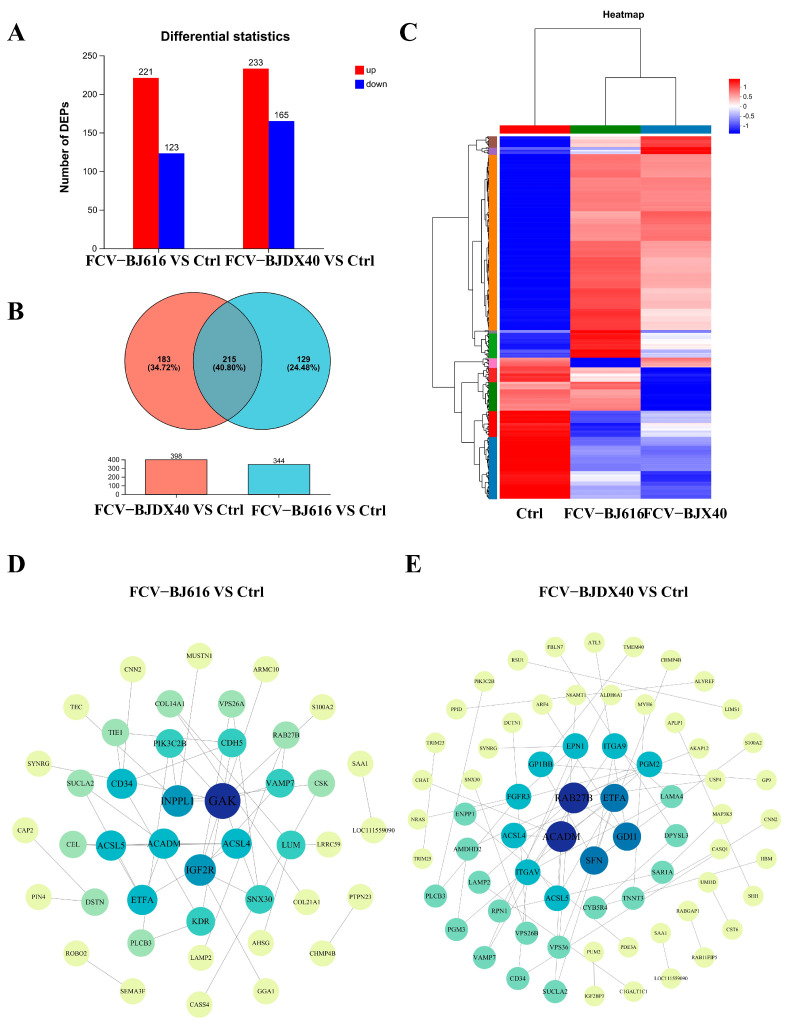
Differential protein hierarchy clustering and protein–protein interaction (PPI) network analysis in cat serum infected with FCV. (**A**,**B**) Statistical analysis of differentially expressed proteins (DEPs) in cat serum from FCV-BJ616 and FCV-BJDX40 infection groups. (**C**) Heatmap visualization of all proteins. DEPs in serum from control (Ctrl) and FCV-BJ616/FCV-BJDX40 infection groups. (**D**,**E**) Cluster analysis of PPI networks for DEPs in cat serum from FCV-BJ616/FCV-BJDX40 infection groups.

Differentially expressed proteins (DEPs) were identified from proteomic read counts using thresholds of |fold change (FC)| > 2 and *p*-value < 0.05, comparing FCV-BJ616 and FCV-BJDX40 infection groups versus the uninfected control (Ctrl). Distinct DEP profiles emerged: FCV-BJ616 infection induced 221 upregulated and 123 downregulated DEPs, while FCV-BJDX40 infection resulted in 233 upregulated and 165 downregulated DEPs (Figure 3A). Venn analysis revealed strain-specific differences, including 183 DEPs exclusive to FCV-BJ616, 129 unique to FCV-BJDX40, and 215 co-modulated DEPs (Figure 3B). Hierarchical clustering confirmed significant expression stratification between infection groups and Ctrl (Figure 3C). Protein–protein interaction (PPI) networks further delineated strain-specific functional modules and interaction topology (Figure 3D,E).

### 2.4. GO and KEGG Enrichment Analyses for DPEs

**Figure 4:**
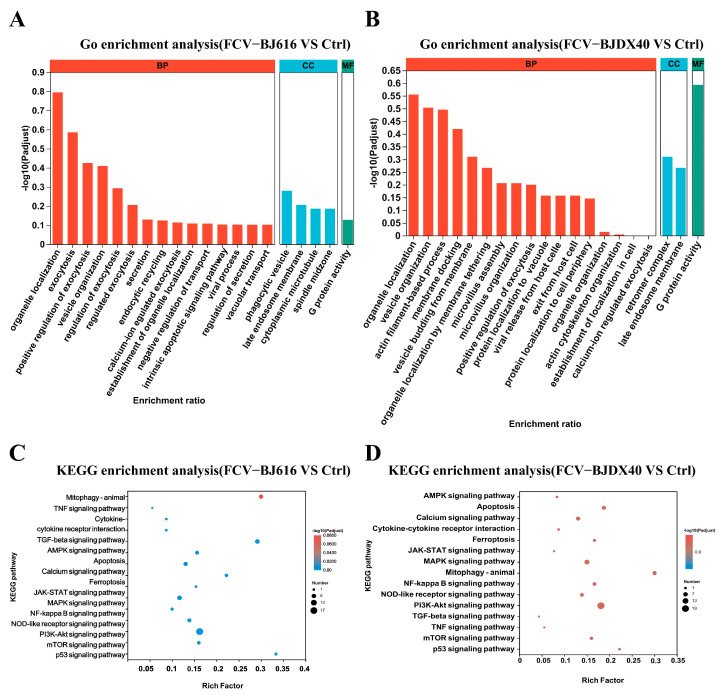
GO and KEGG functional enrichment analysis of DEPs in serum from cats infected with FCV. (**A**,**C**) show the results for cats infected with FCV-BJ616, while (**B**,**D**) present those for cats infected with FCV-BJDX40.

To explore the potential mechanisms of proteins, this study conducted GO and KEGG enrichment analyses. The results of the GO enrichment analysis showed differences in significance and enrichment ratio among biological processes (BP), cellular components (CC), and molecular functions (MF). In the FCV-BJ616 dataset, ‘localization of organic compounds’ was the most significantly enriched, while FCV-BJDX40 exhibited significant enrichment in “actin filament process” (Figure 4A,B). KEGG pathway enrichment analysis revealed that pathways such as “mitotic autophagy—animal”, “TNF signaling pathway”, “NF-kappa B signaling pathway”, “PI3K-Akt pathway”, and “ferroptosis” were enriched to varying degrees with different significance across the datasets (Figure 4C,D). Overall, these analyses revealed similarities and differences between the two datasets in multiple important biological processes and signaling pathways, providing important clues for the biological mechanisms of gene expression.

### 2.5. Analysis of Candidate Biomarkers in DEPs

**Figure 5:**
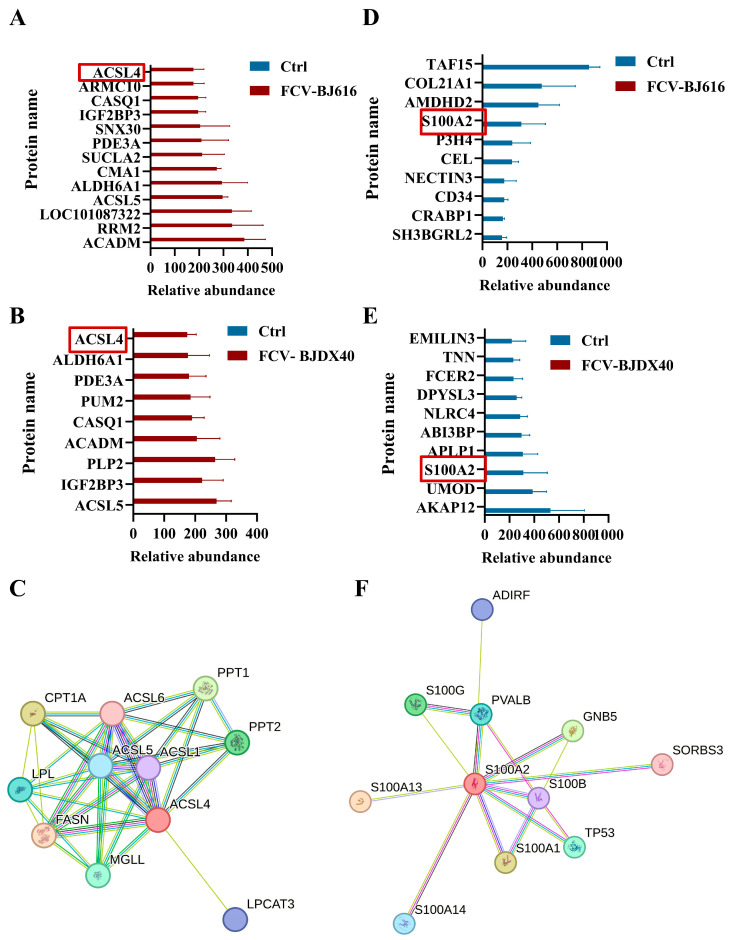
Identification of FCV infection marker genes. (**A**,**B**) ACSL4 serve as upregulated markers for FCV-BJ616 and FCV-BJDX40 infections. (**C**) PPI network of ACSL4 and ACSL5 interacting genes. (**D**,**E**) S100A2 acts as a downregulated marker for FCV-BJ616 and FCV-BJDX40 infections. (**F**) PPI network of S100A2 interacting genes. The red boxes are labeled ACSL4 and S100A2 respectively.

The fold change in signature proteins often reflects their potential as core biomarkers, especially in response to pathological mechanisms and diagnostic applications. This study analyzed proteins that were significantly upregulated or downregulated (Fold change > 8) in serum following FCV-BJ616 and FCV-BJDX40 infections. Our analysis revealed that ACSL4 was significantly upregulated in both serum groups, and this upregulation may be associated with lung injury caused by ferroptosis (Figure 5A–C). Additionally, among the genes significantly downregulated in both groups, S100A2 stood out (Figure 5D–F). The downregulation of S100A2 has been reported to be closely linked to oral diseases, suggesting that S100A2 could serve as a potential prognostic biomarker for FCV.

### 2.6. The Candidate Protein Was Validated by Western Blotting

**Figure 6:**
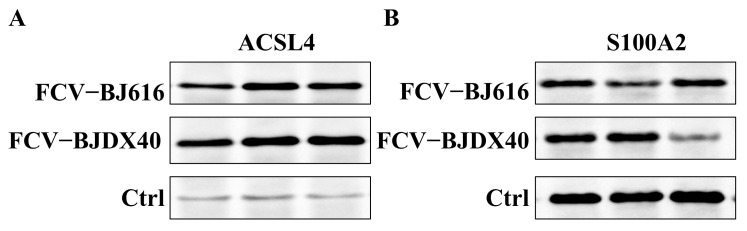
Western blotting analysis of marker expression in cat serum infected with FCV. (**A**) Western blotting analysis of ACSL4 expression in serum from cats infected with FCV-BJ616 and FCV-BJDX40. (**B**) Western blotting analysis of S100A2 expression in serum from cats infected with FCV-BJ616 and FCV-BJDX40.

Western blotting was used to evaluate the expression patterns of ACSL4 and S100A2 in clinical serum samples from healthy controls (Ctrl) and FCV-infected cats. The analysis revealed a significant upregulation of ACSL4 in the FCV group (Figure 6A), while the expression of S100A2 was markedly downregulated (Figure 6B). Collectively, these expression changes demonstrate both consistency with FCV infection and high specificity.

## 3. Discussion

Current detection of FCV infection primarily relies on RT-qPCR and virus isolation from swab samples (preferably oropharyngeal or lingual swabs) [23,24]. However, these methods face challenges, including amplification failures due to high viral genetic variability, degradation from suboptimal sample quality or transport, antibody interference, and the fact that negative results do not reliably rule out infection [25]. Immunodiagnostic techniques such as immunohistochemistry can confirm infection at the tissue level, but their invasiveness limits routine application [25]. Serological testing also struggles to distinguish active from past infection. In particular, for VSD-FCV, no single test reliably predicts disease progression. This diagnostic gap underscores the need for complementary biomarkers that capture real-time host responses—especially markers that quantify tissue injury severity (e.g., lung injury) and inform prognosis. Serum, a practical and dynamic repository of pathophysiological signals, is a suitable matrix for discovering such biomarkers [11,25,26]. Accordingly, we used quantitative proteomics to identify serum biomarkers related to FCV pathogenesis.

We observed significant upregulation of ACSL4 and ACSL5 in serum from FCV-infected animals, suggesting that these long-chain acyl-CoA synthetases are associated with FCV disease progression. Notably, ACSL4 is a key regulator of ferroptosis, an iron-dependent form of programmed cell death driven by lipid peroxidation [27,28,29,30]. This core finding aligns with reports in human sepsis-induced lung injury. Recent studies report that in acute lung injury, diverse upstream mechanisms—including epigenetic modifications (e.g., histone lactylation), signaling proteins (e.g., SHP2), and non-coding RNAs (e.g., circEXOC5)—converge to upregulate ACSL4, thereby promoting iron-dependent lipid peroxidation and alveolar epithelial cell death [31,32,33]. Given the severe lung injury observed in our FCV-infected animals and the established role of ACSL4 in ferroptosis, we hypothesize that FCV-induced upregulation of ACSL4 increases cellular susceptibility to lipid peroxidation. Mechanistically, this may involve promoting the incorporation of specific polyunsaturated fatty acids (e.g., arachidonic acid) into phospholipids, facilitating ferroptotic cell death and contributing to lung pathology. Consequently, the elevated levels of ACSL4 in serum show promise as a candidate biomarker.

Concurrently, we observed persistent downregulation of serum S100A2 in FCV-infected groups. S100A2 supports epithelial integrity, antimicrobial defense, and immune homeostasis [34]. Importantly, prior studies have associated lower S100A2 expression with stomatitis severity [35,36]. Given that oral inflammation is a hallmark of FCV infection [37,38], sustained S100A2 downregulation may contribute to, or reflect, oral inflammatory pathology in FCV. Accordingly, serum S100A2 dynamics may serve as a biomarker to track progression toward chronic FCV infection and to monitor associated immune responses. A combined panel of ACSL4 and S100A2 may provide a more comprehensive view of the host response than either marker alone.

This study has several limitations. First, the small sample size and use of an experimental challenge model necessitate validation in larger cohorts of naturally infected clinical cases. The absence of such cases in the current study limits immediate generalizability to the broader FCV-positive feline population encountered in clinical practice. Second, although we propose serum ACSL4 and S100A2 as a window into tissue pathology, we did not perform direct tissue–serum correlation analyses. Thus, the cellular origins of these serum biomarkers and their quantitative correlations with lung histopathology remain to be elucidated. Future studies incorporating parallel tissue proteomics and histopathology will be crucial to confirm mechanistic roles and to establish diagnostic value. Acknowledging these points, we position this work as a discovery-phase study that provides a strong rationale and clear direction for subsequent validation and mechanistic investigation.

In conclusion, quantitative proteomics identified significant FCV-induced alterations in the host serum proteome. The upregulation of ACSL4 is potentially associated with ferroptosis-mediated lung injury, whereas the downregulation of S100A2 may have prognostic value. These insights advance understanding of FCV pathogenesis, particularly with respect to lung injury, and lay the groundwork for diagnostic tools, prognostic biomarkers, and ferroptosis-targeted interventions.

## 4. Materials and Methods

### 4.1. Cells and Viruses

CRFK (Crandell-Rees Feline Kidney) cells were cultured in Dulbecco’s modified Eagle medium (DMEM; Gibco, Waltham, MA, USA) supplemented with 10% fetal bovine serum (FBS; Gibco, USA) and maintained at 37 °C under 5% CO_2_ in a humidified incubator. The FCV-BJ616 (CGMCC No. 47081) and FCV-BJDX40 (CGMCC No. 47082) strains used in this study were isolated from feline nasal swabs of cases with severe respiratory symptoms at Beijing Animal Hospital in 2024.

### 4.2. Indirect Immunofluorescence Assay (IFA)

CRFK cells were seeded in 24-well plates and subsequently infected with strains BJ616 and BJDX40 at a multiplicity of infection (MOI) of 1. After 24 h post-infection (hpi), the supernatant was removed, and cells were fixed with 4% paraformaldehyde for 30 min at room temperature (RT). Following fixation, cells were permeabilized with 0.1% Triton X-100 in PBS for 10 min, then washed thrice with PBS. Subsequently, cells were blocked with PBS containing 2% bovine serum albumin (BSA) for 30 min at 37 °C. After blocking, cells were incubated with anti-FCV-VP1 protein antiserum (in-house prepared) at 37 °C for 1 h, followed by three washes with PBS. After washing, cells were incubated with a goat anti-rabbit immunoglobulin G (IgG) (H+L) cross-adsorbed secondary antibody conjugated to Alexa Fluor 488 (Thermo Fisher Scientific, Waltham, MA, USA) for 45 min. Following another washing step, nuclei were counterstained with 4′,6-diamidino-2-phenylindole (DAPI; Roche, Basel, Switzerland) for 3 min. Finally, cells were washed thrice with PBS and imaged using a fluorescence microscope.

### 4.3. Animal Experimentation

This study used 9 healthy 3-month-old cats. These cats were provided by the Institute of Animal Science and Veterinary Medicine, Chinese Academy of Agricultural Sciences. Experimental cats were housed in an environment with suitable temperature and humidity (20–26 °C, 40–70%), good lighting and ventilation. Each cage had an area of ≥0.8–1.2 m^2^ per cat. Appropriate diet was provided, and veterinarians regularly monitored disease prevention and control. New cats were quarantined, and the cages were furnished to meet their behavioral needs. They were not infected with feline herpesvirus, feline panleukopenia virus, or feline coronavirus, and did not produce FCV antigens or antibodies. Following a two-week acclimatization period, cats were randomly assigned to three groups (*n* = 3 per group): (1) infected with strain BJ616, (2) infected with strain BJDX40, and (3) uninfected negative control. Cats in infection groups received 500 µL of either BJ616 (1.0 × 10^8.5^ TCID_50_/mL) or BJDX40 (1.0 × 10^8.2^ TCID_50_/mL) administered via the ocular, intranasal, and oral routes. Control cats received an equivalent volume of sterile vehicle (cell culture medium) via the same routes. Following infection, cats were monitored daily for rectal temperature, body weight, clinical signs, and survival.

The clinical status scoring criteria were as follows (1 point awarded for any of the following): 1. Fever (temperature >39 °C); 2. Anorexia; 3. Drowsiness; 4. Weight loss (>5%); 5. Inability to stand, severe respiratory distress, or near death [39]. If a score of 4 was reached, samples were allowed to clot overnight at 4 °C, then centrifuged at 3000× *g* for 10 min at 4 °C to separate serum. The serum supernatant was collected, aliquoted, flash-frozen in liquid nitrogen, and stored at −80 °C until analysis. During the experiment, feline subjects that lost the ability to eat, drink, and developed respiratory distress were euthanized. Humane endpoints were strictly observed. Cats exhibiting severe clinical signs indicating irreversible suffering or meeting predefined euthanasia criteria (e.g., inability to eat/drink, severe respiratory distress, moribund state) were euthanized by intravenous barbiturate overdose following Chinese Academy of Agricultural Sciences IACUC protocols. Necropsy was performed immediately after euthanasia. Liver, kidney, lung, and spleen tissues were collected and fixed in 4% paraformaldehyde. Fixed tissues were processed routinely, embedded in paraffin, sectioned, and stained with hematoxylin and eosin (H&E) for histopathological examination of lesions, particularly in the lungs.

### 4.4. Serum Protein Extraction and Trypsin Digestion

The research methodology was referenced from prior literature [40,41]. Frozen serum samples were thawed on ice. Samples were homogenized in protein lysis buffer (8 M urea, 1% SDS) supplemented with protease inhibitor cocktail using a high-throughput tissue homogenizer (3 cycles of 180 s each). Subsequently, homogenates were subjected to non-contact ultrasonication on ice for 30 min. After centrifugation at 16,000× *g* at 8 °C for 30 min, the resulting supernatant was collected. Protein concentration was determined using the bicinchoninic acid (BCA) assay with a commercial kit (Thermo Scientific) according to the manufacturer’s instructions. Protein quantification was performed following the kit’s protocol. Following quantification, protein samples (100 μg per sample) were denatured and reduced by suspension in 100 mM triethylammonium bicarbonate (TEAB) buffer containing 10 mM tris(2-carboxyethyl)phosphine (TCEP) and incubation at 37 °C for 60 min. Subsequently, cysteine residues were alkylated by adding iodoacetamide (IAM) to a final concentration of 40 mM and incubating at room temperature in the dark for 40 min. After alkylation, proteins were precipitated using acetone to remove detergents and salts. The protein pellet was washed, air-dried briefly, and then dissolved/reconstituted in 100 μL of 100 mM TEAB buffer. Trypsin digestion was performed at a 1:50 (trypsin:protein, *w*/*w*) ratio overnight (~16 h) at 37 °C. After trypsin digestion, the peptides were dried using a vacuum pump. The enzymatically processed peptides were then re-solubilized with 0.1% trifluoroacetic acid (TFA), desalted with HLB, and dried using a vacuum concentrator. Finally, the peptides were quantified using the NANO DROP ONE (Thermo Scientific) by measuring UV absorption.

### 4.5. DIA Mass Detection

LC-MS/MS analysis was performed using a Vanquish Neo UHPLC system coupled online to an Orbitrap Astral mass spectrometer (Thermo Fisher Scientific) at a core facility. The uPAC High Throughput column (75 µm × 5.5 cm, Thermo, Waltham, MA, USA) was used with solvent A (water containing 2% ACN and 0.1% formic acid) and solvent B (water containing 80% ACN and 0.1% formic acid). The chromatography run time was set to 8 min. Data were acquired using data-independent acquisition (DIA) with an Orbitrap Astral mass spectrometer operated in DIA mode. The mass spectrometry scanning range was from 100 to 1700 *m*/*z*. Spectronaut software (Version 19) was used to analyze the DIA raw data. The parameters were as follows: peptide length range set to 7–52; enzyme cutting site was trypsin/P; maximum missed cleavage site was 2; carbamidomethylation of cysteines as a fixed modification, and oxidation of methionines and protein N-terminal acetylation as variable modifications; protein FDR ≤ 0.01, peptide FDR ≤ 0.01, peptide confidence ≥ 99%, XIC width ≤ 75 ppm. The protein quantification method used was MaxLFQ [42].

### 4.6. Bioinformatics Analysis

Bioinformatic analysis of the proteomic data was conducted using the Majorbio Cloud platform (https://cloud.majorbio.com, accessed on 30 December 2025). The *p*-values and fold change (FC) for the proteins between the two groups were calculated using the R package (version 4.3.3) “*t*-test”. Thresholds for fold change (>2 or <0.5) and *p*-value (<0.05) were applied to identify differentially expressed proteins (DEPs). Functional annotation and enrichment analysis of all quantified proteins and DEPs were performed based on Gene Ontology (GO) terms and Kyoto Encyclopedia of Genes and Genomes (KEGG) pathways. Protein–protein interaction (PPI) networks were constructed using the STRING database (version 11.5) [43].

### 4.7. Western Blotting

Serum protein concentrations were measured using the Pierce BCA Protein Assay Kit (Thermo Fisher). Equal amounts of protein (30 µg) were separated by SDS-PAGE and transferred onto polyvinylidene fluoride (PVDF) membranes. Membranes were blocked with TBST (Tris-buffered saline with 0.1% Tween-20) containing 5% non-fat dry milk for 2 h at room temperature, then incubated with primary antibodies against ACSL4 (1:1000; HY-P80977, MedChemExpress, Monmouth Junction, NJ, USA) and S100A2 (1:1000; D227499-0025, Sangon Biotech, Shanghai, China) diluted in blocking buffer overnight at 4 °C. After washing three times with TBST, membranes were incubated with horseradish peroxidase (HRP)-conjugated secondary antibody (1:10,000) in blocking buffer for 1 h at room temperature. Following three additional TBST washes, protein bands were detected using the ECL Prime Western Blotting Detection Reagent (GE Healthcare, Hertfordshire, UK) according to the manufacturer’s instructions.

## 5. Conclusions

This study confirms the widespread prevalence of Feline Calicivirus (FCV) in China and its significant cytopathic effects. Proteomic analysis revealed that FCV infection induces extensive dysregulation of host protein expression, primarily by perturbing key pathways including TNF signaling and ferroptosis. The differential expression of ACSL4 and S100A2 in serum was further verified by Western blotting. Consequently, ACSL4 and S100A2 emerge as promising biomarkers for monitoring FCV infection and disease progression, laying a groundwork for the future development of diagnostic and prognostic tools.

## Data Availability

The mass spectrometry proteomics data have been deposited at the ProteomeXchange Consortium via the PRIDE [44] partner repository with the dataset identifier PXD070490. URL: https://www.ebi.ac.uk/pride/archive?keyword=PXD070490 (accessed on 30 December 2025).
